# *Invivo*Pen: A novel plasma source for *in vivo* cancer treatment

**DOI:** 10.7150/jca.38613

**Published:** 2020-02-10

**Authors:** Xin Zhou, Dongyan Cai, Shaoqing Xiao, Meng Ning, Renwu Zhou, Shuo Zhang, Xiao Chen, Kostya Ostrikov, Xiaofeng Dai

**Affiliations:** 1 Engineering Research Center of IoT Technology Applications (Ministry of Education), Department of Electronic Engineering, Jiangnan University, Wuxi, China; 2 Wuxi School of Medicine, Jiangnan University, Wuxi, China; 3 Department of Oncology, Affiliated Hospital of Jiangnan University, Wuxi, China; 4 School of Mechanical Engineering, Jiangnan University ,Jiangsu Wuxi 214122, China; 5 Laboratory of Advanced Food Manufacturing Technology of Jiangsu Province, Jiangnan University, Jiangsu Wuxi 214122, China; 6 School of Biotechnology, Jiangnan University, Wuxi, China; 7 School of Chemistry, Physics and Mechanical Engineering, Queensland University of Technology, Brisbane, Queensland 4059, Australia; 8 Institute of Health and Biomedical Innovation, Queensland University of Technology, Brisbane, Queensland 4059, Australia; 9 The First Affiliated Hospital of Xi'an Jiaotong University, Xi'an, 710061, China

**Keywords:** triple negative breast cancer, plasma jet, *invivo*Pen, plasma activated medium, cancer treatment

## Abstract

**Background**: With the anti-cancer efficacies of cold atmospheric plasma being increasingly recognized *in vitro*, a demand on creating an effective tool feasible for *in vivo* animal treatment has emerged.

**Methods**: Through the use of co-axial needles with different calibers in diameter, we designed a novel *in situ* ejection source of cold atmospheric plasma, namely *invivo*Pen, for animal experiments. It punches just a single pinhole that could considerably ease the complexity of operating with small animals such as mouse.

**Results**: We showed that *invivo*Pen could deliver similar efficacies as plasma activated medium with reduced cost in suppressing cell proliferation and migration as well as potentially boosting the viabilities of mice receiving *invivo*Pen treatment. Blood test, renal and liver functionalities tests all suggest that physical plasma could effectively return tumor-carrying mice to the healthy state without harm to body conditions, and invivoPen slightly outweighs PAM in boosting animal immunity and reducing inflammation.

**Conclusion**: Our study contributes to the community in providing a minimal invasive *in situ* plasma source, having partly explained the efficacies of cold atmospheric plasma in treating triple negative breast cancers, and proposing the potential synergies between physical plasma and conventional drugs for cancer treatment.

## Introduction

Physical plasma is the fourth state matter following solid, liquid and gas. It is an ionized gaseous electroneutral material composed of positively charged ions, electrons and neutral particles 1. Physical plasma can be artificially generated by subjecting a neutral gas to a strong electromagnetic field to the point where ionized gaseous substances become increasingly electrically conductive. Cold atmospheric plasma (CAP) refers to non-equilibrium ionized gases under atmospheric pressure, with the temperature being, in general, between 25°C and 45°C 2.

CAP has been widely adopted in many biological applications such as disinfection/ sterilization 3, chronic wound healing and ulceration treatment 4. Ever since the first report on the anti-cancer property of CAP in 2007 5, its potential as an emerging oncotherapy has been gaining increased attention, with efficacies observed in cells of many tumors including, e.g., brain 6, skin 7, breast 8, colorectal 9, lung 10, 11, cervical 12, leukemia 13, liver 14, and head and neck 15, 16 cancers.

Typical configuration of include dielectric barrier discharge (DBD), plasma jet, and plasma torch 17. One of the commonly used medically-relevant plasma source is kINPen MED 18 which, however, cannot penetrate through the skin and can only be used to treat cells or surface diseases. Mirpour et al. have established a micro-sized plasma jet for *in vivo* animal treatment 19 that requires an extra hole in the tissue for exhaust gas exhalation. However, in most cases, tumors grown in animal models such as mouse are small (<1 cm in diameter) and fragile; thus building a gas path within tumors might impose extra challenges to people who conduct the experiments. Also, if applied in clinics, generating two holes for a gas flow within solid tumors may create too invasive to patients that largely reduces their life qualities before achieving any desirable CAP therapeutic efficacy. As far as we know, *in vivo* CAP treatment is largely achieved using plasma activated medium (PAM) that preserves the activities of CAP within a short period of time such as a week 20, 21, and no *in vivo* CAP ejection tool with minimal invasion is so far available.

We found from our previous studies that CAP can selectively target triple negative breast cancer (TNBC) cells both *in vitro* and *in vivo*, a subtype of breast cancers lacking effective therapeutic modalities with little side effect. We are motivated to establish an* in vivo* CAP ejection platform feasible for solid tumor treatment to further investigate the therapeutic efficacies of CAP in treating TNBC tumors *in vivo*. We designed a CAP ejection source for minimal invasive *in situ* treatments, namely *invivo*Pen, and demonstrated that it could convey similar efficacies in cancer proliferation and migration control but more favorable effects in preserving mice viabilities than PAM in treating TNBC-inoculated mice. We also proposed that *invivo*Pen delivered its selective therapeutic efficacies on TNBCs through inducing luminal-like features of such cancers and selectively targeting cancer stem cells.

## Materials and methods

### Cell culture

A human breast cancer cell line, MDA-MB-231, purchased from American Type Culture Collection (Manassas, VA, USA), was used in this study. The cell line was cultured following supplier's recommendation. Cells were grown at 37 °C and 5% (v/v) CO_2_ in a humidified incubator. All media were purchased from Hyclone Laboratories, Inc. (United States).

### Characterization of* invivo*Pen

The temperatures of *invivo*Pen at different peak-to-peak voltages were measured by the thermometer attached to the *invivo*Pen outlet. A human impedance analog circuit 22 (Figure 2A) was used to simulate the current and voltage of *invivo*Pen when it contacts with human body. The electrical properties of *invivo*Pen were measured in 'skin resistance' given that *invivo*Pen functions by puncturing through the skin. AC (Alternating Current) contact current and AC contact voltage between nodes A and B was measured by a multimeter.

UV-visible-NIR, with the wavelengths ranging from 200 nm to 800 nm, of the CAP generated by *invivo*Pen was measured using Andor's Mechelle ME5000 spectrograph and iStar334T ICCD to detect reactive species such as nitrogen [N2], nitric oxide [-NO], [N+2], atomic oxygen [O], and hydroxyl radical [-OH].

### Optical emission spectra measurement

The spectrometer and the detection probe were purchased from Andor Technology. The optical probe was placed 2 cm above the plasma jet nozzle. Integration time for data collection was set to 100 ms, frequency was set to 8.8 kHz, peak-to-peak output electrode voltage was set to 11 kV, and gas flow was controlled at 1 L·min^-1^.

### *In vivo* tumor treatment

All animal experiments were performed in accordance with the National Institutes of Health Guide for the Care and Use of Laboratory Animals and were approved by the Animal Laboratory Center of Jiangnan University.

3×10^6^ MDA-MB-231 cells suspended in 100 μL phosphate buffer solution (PBS) were injected subcutaneously in the right forelimbs of 16 female BALB/c mice aged 3-4 weeks with the weights of 16 ± 2 g on the first day. 15 MDAMB231 cell inoculated BALB/c mice were evenly divided into 3 groups, i.e., *invivo*Pen group (receiving *invivo*Pen treatment), PAM group (receiving PAM injection), and null group (no treatment). The first treatment was performed when tumor sizes reached 5 ± 0.5 mm, which were calculated following Equation 1:


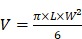
 Equation 1

where '
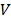
', '
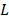
' and '
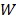
' each represents the volume, largest diameter and smallest diameter of the tumor, respectively. Tumor diameters were measured using vernier caliper. Mice were anesthetized with ketamine (concentration is 10 mg·ml^-1^) intraperitoneally before each treatment. The injection volume was 10 μl·g^-1^ of the mouse body weight. Two approaches '*invivo*Pen treatment' and 'PAM injection' were conducted following procedures below:

*invivo*Pen treatment: a mouse was paralyzed following fixation on its back onto a liftable-insulation board. The generator of *invivo*Pen was turned on, the peak-to-peak electrode voltage was set to 5 kV, the sinusoidal wave frequency was set to 8.8 kHz, and the Helium gas flow rate was set to 0.2 L·min^-1^. The nozzle of *invivo*Pen was punched into mouse tumor through lifting the insulation board following 5 min CAP exposure. The heartbeat of mouse was monitored throughout the treatment process.

PAM injection: PAM was prepared by fixing the distance between *invivo*Pen and the medium surface as 13 mm and treating each well of a 12-well plate for 10 min where each well was filled with 2 ml PBS. Parameters were set the same as in '*invivo*Pen treatment' and in optical emission spectra measurement. PAM was subcutaneously injected at two slots of the tumor for each mouse with 100 uL/slot following the same animal operation procedure as in '*invivo*Pen treatment'.

These treatments were repeated every 72 hours for each mouse until its death or the end of this study (30 days). Tumors were dissected after the sacrifice of the mice.

### Animal blood test

Mice were intraperitoneally anesthetized before the blood was taken each time using 150μL 1% pentobarbital sodium solution. Four drops of blood were taken using a capillary tube through inserting into the mouse eye from the inner canthus and puncturing the posterior orbital vein. The blood was stored in centrifugal tubes infiltrated with 0.1% heparin sodium to prevent blood clotting. The blood routine analysis was operated by Mindray automatic blood analyzer.

Before the sacrifice of the mice, eyeball extraction was operated to collect blood for liver and renal functionality tests. For each mouse, the blood was placed in centrifuge tubes for 4 h following centrifugation at 3000 rpm for 10 min, and the extracted serum was used to test liver and renal functions. Indexes of liver and renal functions were measured by Mindray fully automatic biochemical analyser.

### Immunohistochemistry staining (IHC)

The 5 μm thick paraffin sections were deparaffinized in xylene and rehydrated in ethanol at different gradients (100%, 100%, 95%, 70% in sequence). Tissue slices were incubated in 3% H_2_O_2_ for 20 min to inactivate endogenous peroxidase. After being heated in 10 mM citrate buffer for 15 min, tissue sections were incubated with primary antibodies ER (21244-1-AP, 1:200; Proteintech, Rosemont, IL, USA), HER2 (60311-1-Ig, 1:200; Proteintech), ALDH1 (#54135, 1:200; Cell Signaling Technology, St Louis, MO, USA) and E-Cadherin (#14472, 1:200; Cell Signaling Technology) overnight at 4°C. Corresponding secondary antibody (#8114; Cell Signaling Technology) was added and incubated for 1 hour at the room temperature. Images were observed with Pannoramic MIDI (3DHISTECH Ltd, Budapest, Hungary).

### TUNEL assay

TUNEL assay was detected using the One Step TUNEL Apoptosis Assay Kit (C1086, Beyotime Biotechnology; Shanghai, PRC). After being deparaffinized, tissue slices were incubated at 37°C for 25 min in none-DNase proteinase-K working liquid (20μg·ml^-1^). TUNEL detection solution was added on the tissue slices and incubated at 37°C for 1 hour following dehydration. After dehydration, the tablet was sealed with the Antifade Mounting Medium with DAPI (H-1200, VECTOR Laboratories; Burlingame, CA). Samples were observed under a fluorescence microscope (ZEISS, Axio Imager Z2; Oberkochen, GER), where the green and blue fluorescence were observed under a 520 nm and a 460 nm laser, respectively.

## Results

### Configuration of* Invivo*Pen

**Figure 1:**
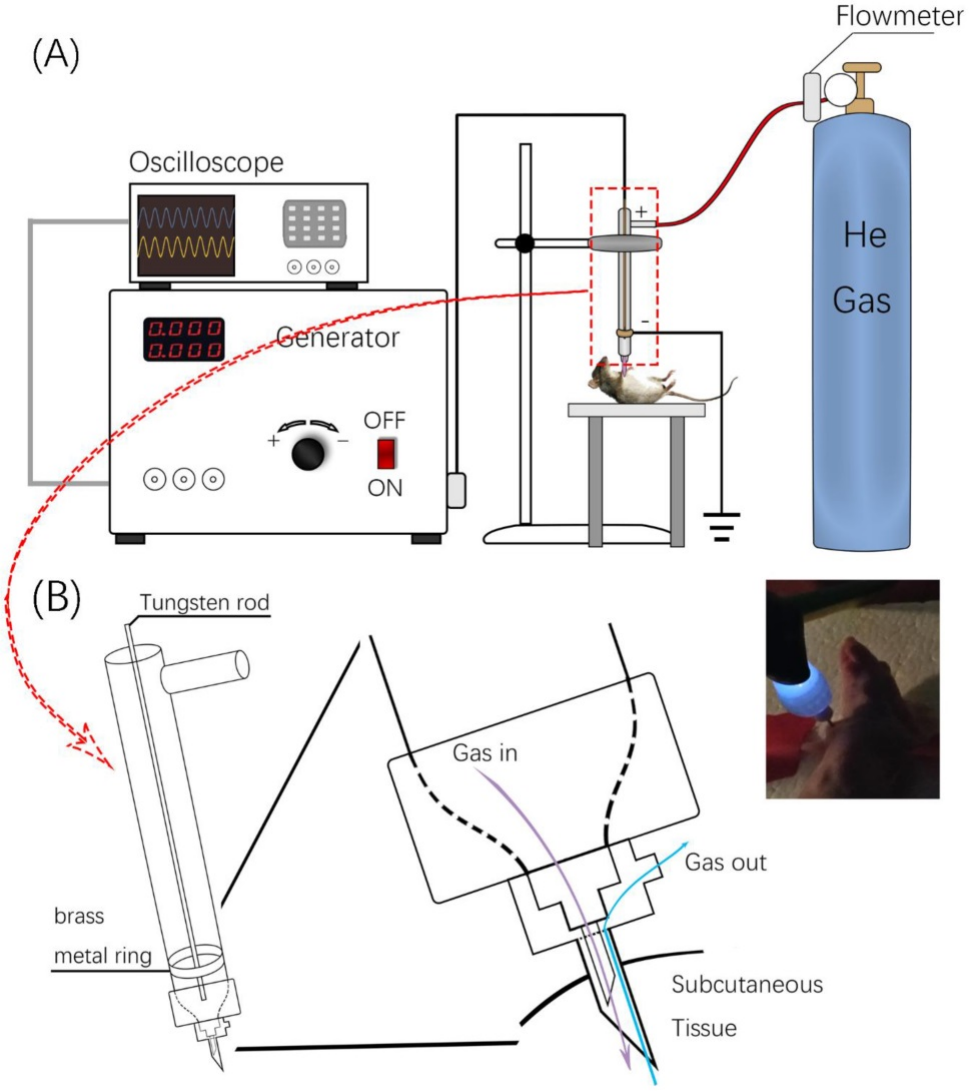
** Design and usage of *invivo*Pen.** (**A**) Schematic illustration of *invivo*Pen design. (**B**)* In situ* mouse treatment using *invivo*Pen.

The *invivo*Pen was developed as a dielectric block discharge (DBD) based CAP device for *in vivo* ejection, which is comprised of a high-voltage radio frequency power supply plasma generator (Coronalab, CTP-2000K), a Helium rotameter (KROHNE), and an oscilloscope (Tektronix, TBS 1102B), connected by two gas pipes, two high voltage wires, and a custom quartz tubes as the blocking medium (Figure 1).

The inner and outer diameters of the quartz tube are 3 mm and 9 mm, respectively. The length of *invivo*Pen is 150 mm. A gas inlet with 30mm in length is 30 mm away from the upper end. The high voltage electrode is a tungsten steel rod with 1mm in diameter, and is fixed along the axis of the quartz dielectric tube in the middle. The negative electrode is a brass metal ring wrapping around the quartz tube, which is located 10 mm above the bottom, and the positive electrode is the tungsten rod inside the quartz tube. The space between the positive and negative electrodes is 1 mm (Figure 1A).

To execute a minimally invasive intratumoral treatment, a unique design of two co-axial needles with different calibers was fabricated in the pinhead. The diameters of the inner and outer needles are 0.25 mm and 0.5 mm, respectively, with the inclined face of the inner needle being in the opposite direction with that of the outer needle. The space in-between the inner and outer needles is the outlet for exhaust gas (Figure 1B).

### Characterization of* invivo*Pen

**Figure 2:**
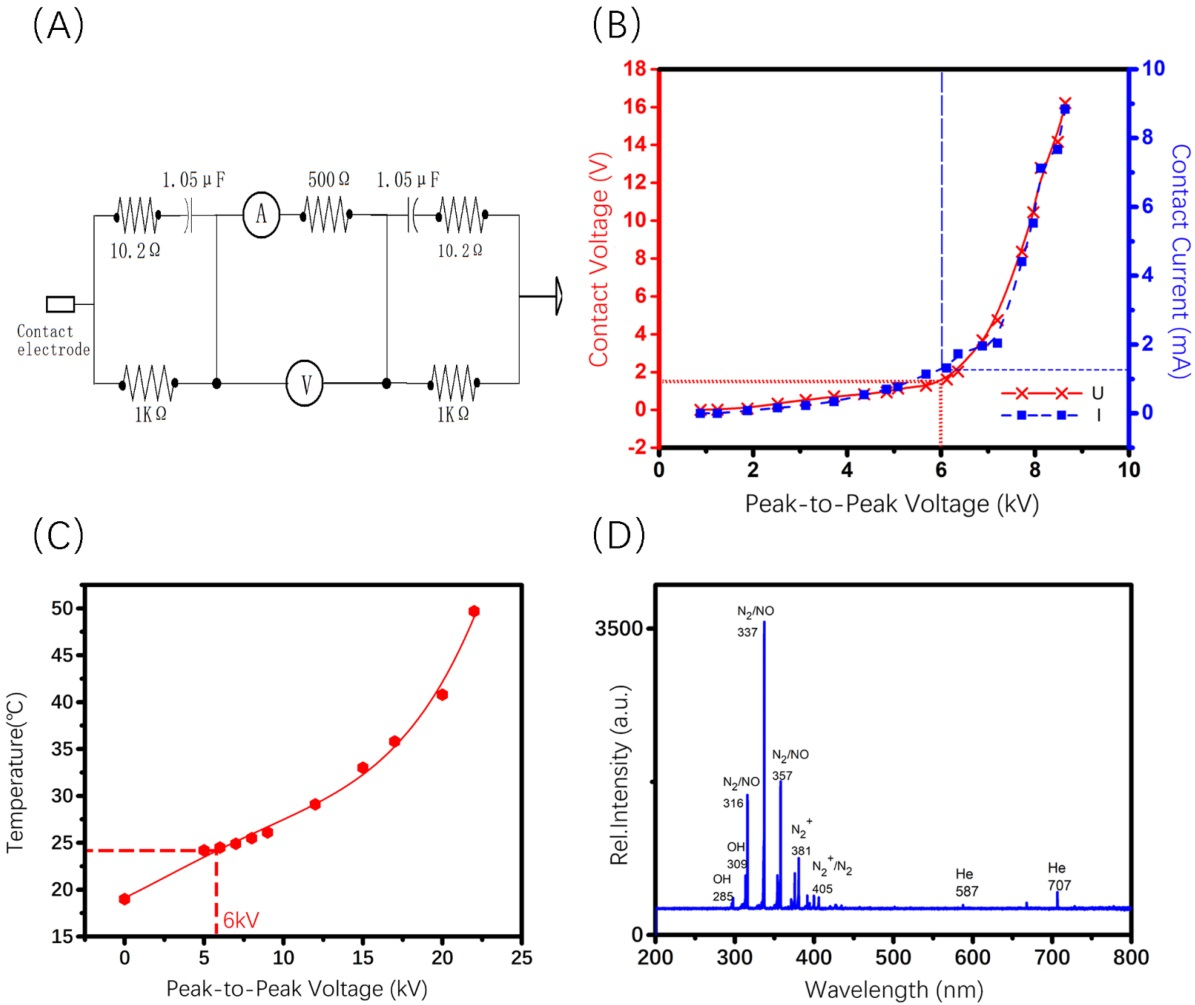
** Charaterization of the components generated using *invivo*Pen.** (A) Human body impedance analog circuit, (B) Electrical characterization, (C) operating temperature examination, and (D) Optical emission spectra analysis of *invivo*Pen.

To maximize the treatment efficacy while ensuring the safety, the Peak-to-Peak voltage was set to 6 kV for *invivo*Pen in the experiments throughout all* in vivo* experiments. The AC contact current was below 2 mA under 6 kV Peak-to-Peak voltage, which is safe to human 23; and the AC contact voltage was below 2 V (Figure 2B), much lower than the International Electrotechnical Commission standard voltage, i.e., 50 V (IEC 60038-2009). Accordingly, the temperature at this voltage was below 30 °C (Figure 2C), which generated no burn. The Peak-to-Peak voltage was between 10 to 13 kV when generating PAM according to 24.

Reactive species produced from *invivo*Pen were comprised of largely nitrogen oxides and hydroxide ions, with nitrogen oxides being the most abundant and almost no helium ions (Figure 2D).

### Characterization of treatment efficacy of *invivo*Pen

**Figure 3:**
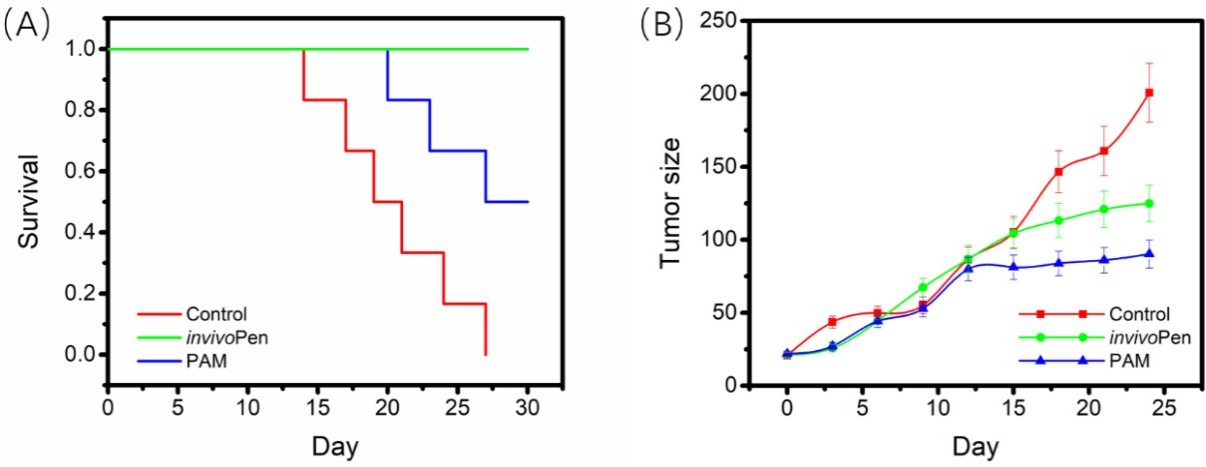
** Comparisons on *invivo*Pen and PAM treatments in preserving mice viability and suppressing tumor growth.** (A) The 30-day survival curves of nude mice after receiving CAP treatment. (B) The 30-day tumor growth curves after CAP treatment.

All mice in the control group died within 27 days, 3 out of 5 mice from the TNBC-PAM group died within 30 days, and all mice from the TNBC-*invivo*Pen group survived to the last day with relatively good viabilities. The 30-day survival of mice in the TNBC-*invivo*Pen group was significantly higher than that of the TNBC-PAM group (p=4.9E-4, Figure 3A). Both *invivo*Pen and PAM significantly inhibited tumor growth with statistical significance (p=0.044 for *invivo*Pen, p=0.017 for PAM), yet the growth of tumors in the TNBC-PAM group was more suppressed than that in the* invivo*Pen group (Figure 3B).

**Figure 4:**
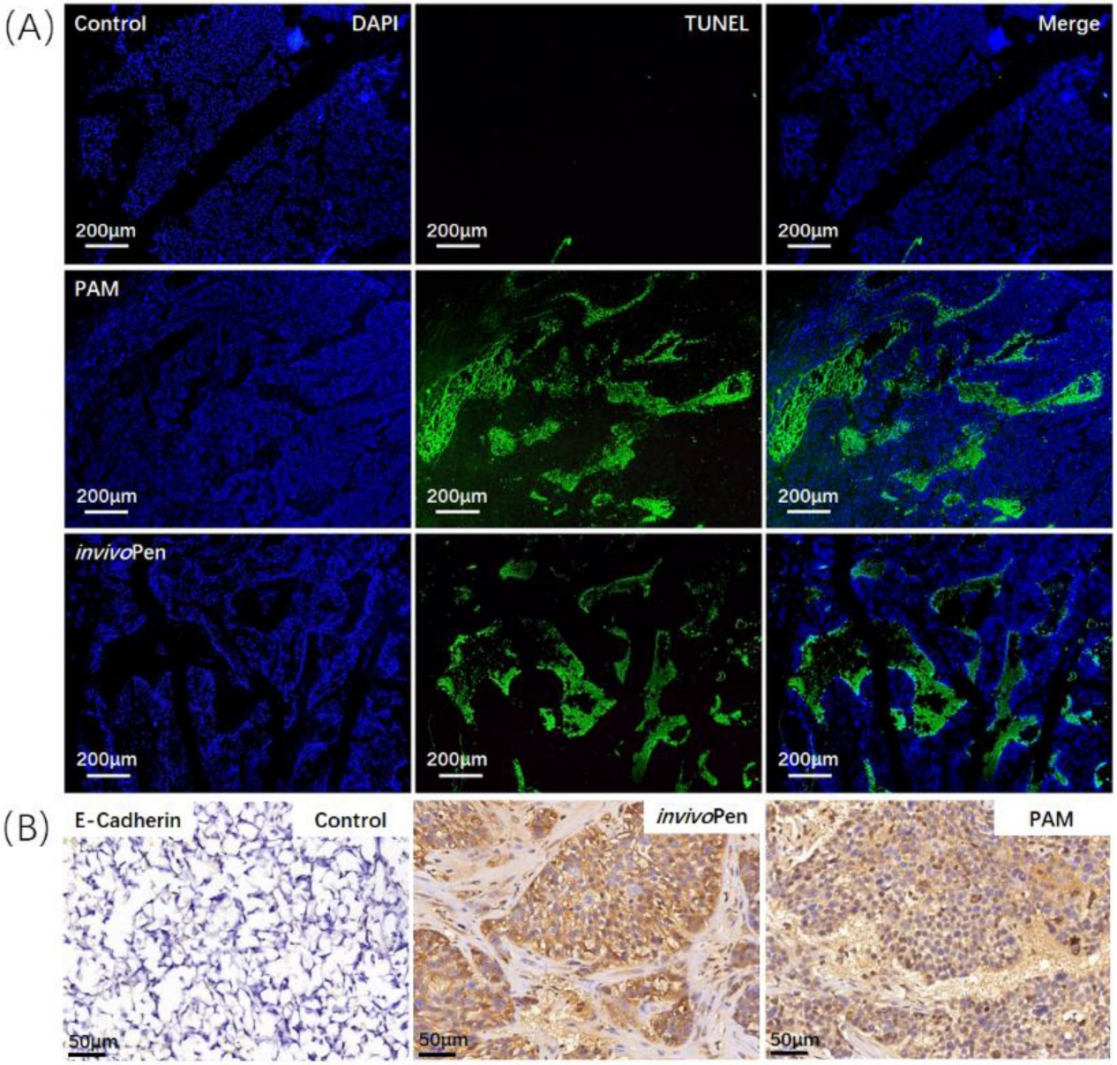
** Comparisons on *invivo*Pen and PAM treatments in inducing *in vivo* tumor apoptosis and reducing tumor migrative abilities.** (A) Tumor cell apoptosis examined using TUNEL staining. (B) Tumor migrative ability examined using immunohistochemistry staining of tumor migration suppressor E-Cadherin. These assays were conducted on tumor samples from mice inoculated with TNBC cells MDBMA231.

Both PAM and *invivo*Pen treatments showed similar efficacies in inducing tumor cell apoptosis and suppressing tumor migrative abilities. A large number of cells undergone apoptosis in PAM- or *invivo*Pen-treated tumor samples (Figure 4A). E-cadherin expression, a clinical immunohistochemistry marker with suppressive role on tumor migration 25, 26, was considerably induced in PAM or *invivo*Pen-treated tumor samples (Figure 4B).

### Safety assessment of *invivo*Pen

**Figure 5:**
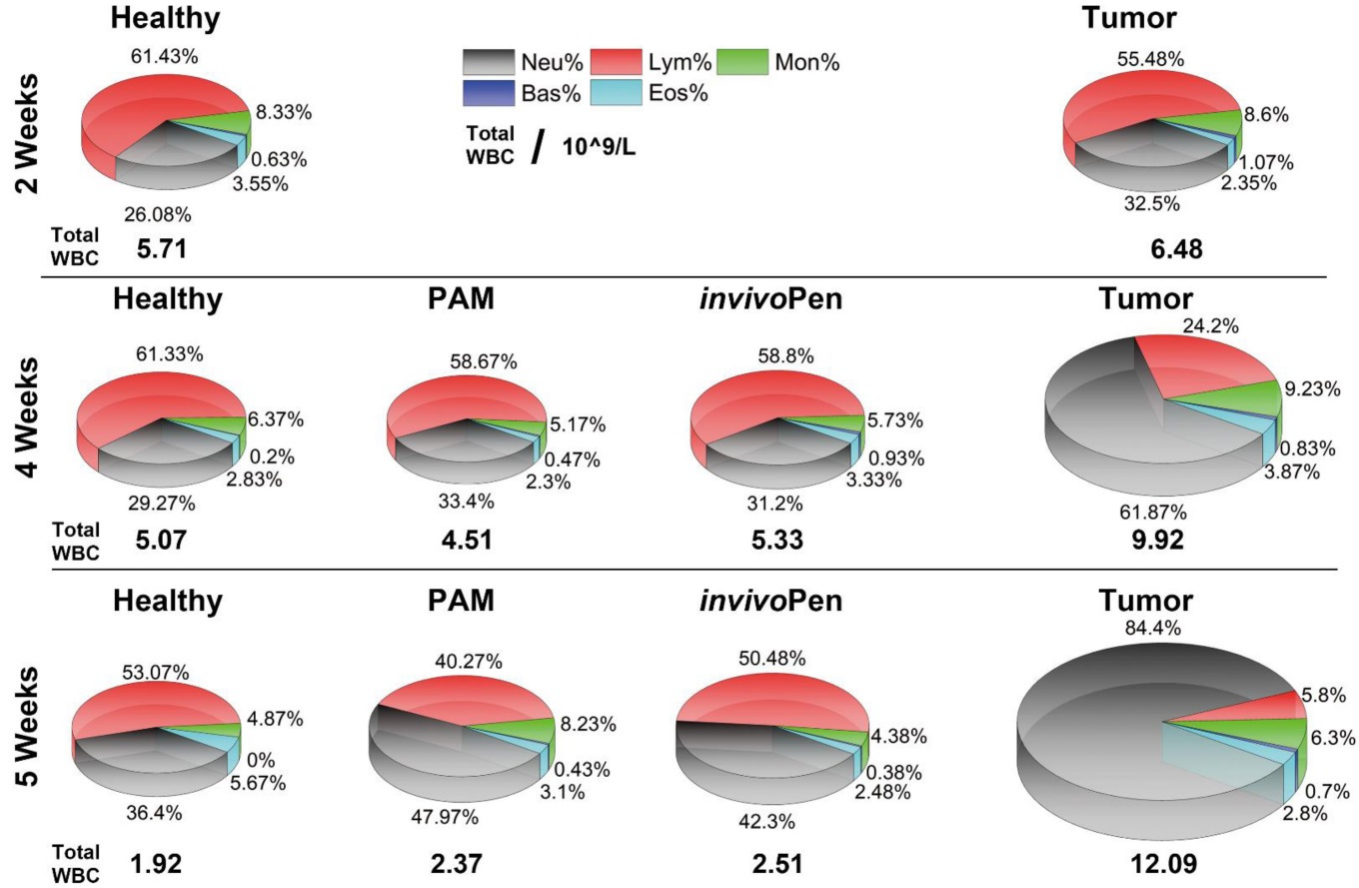
Comparisons on white blood cells between healthy, tumor and tumor receiving *invivo*Pen and PAM treatment *in vivo* at different testing time points.

Blood test results showed that different types of white blood cells were all considerably altered in mice inoculated with tumor cells, and such differences were amplified with time (i.e., from 1.13 times at 2 weeks to 1.95 times at 4 weeks, and to 6.33 times at 5 weeks for the total while blood cells,** Table 1**), and indexes on red blood cells did not alter with statistical significance between healthy and tumor-carrying mice (**Supplementary Table 1**). Both PAM and *invivo*Pen could effectively reduce the total white blood cell level and the fractions of different white blood cell types back to normal (**Figure 5**), and *invivo*Pen slightly outweighs PAM in a few indexes such as monocytes, neutrophil granulocyte, eosinophil granulocyte, and basophil granulocyte (**Table 1**).

Creatine, uric acid, and urea (tested at the 5 weeks) were considerably increased in mice inoculated with tumor cells (**Table 2**), suggesting a damage on renal functionalities when tumor developed. PAM and *invivo*Pen could both effectively reduce levels of these indexes back to normal (**Table 2**).

Indexes on liver functionalities such as alanine aminotransferase, aspartic transaminase, alkaline phosphatase, gamma-glutamyltransferase did not show significant between-group alterations but exhibited large within-group fluctuations (**supplementary Table 2**).

### Characterization of the mechanism leading to CAP treatment efficacies

**Figure 6:**
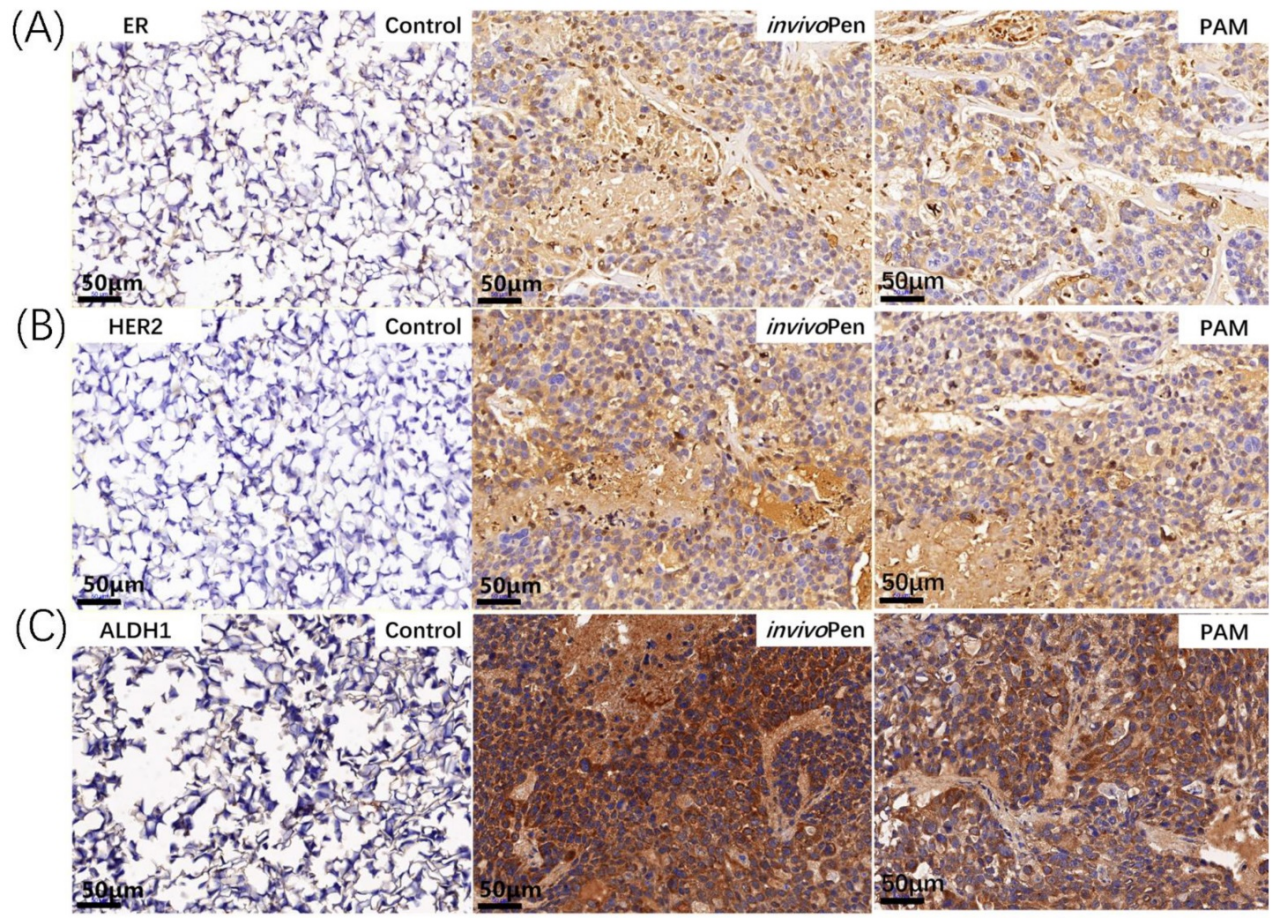
** Immunohistochemistry staining of breast cancer subtyping and cancer stem cell markers in tumor samples.** (A) ER and (B) HER2 are canonical breast cancer subtyping markers, and (C) ALDH1 is a typical cancer stem cell marker. The staining was conducted on tumor samples from mice inoculated with TNBC cells MDBMA231.

Both PAM- and *invivo*Pen- treated TNBC tumors showed increased expression of ER and HER2 (Figure 6A, 6B), which are two primary canonical markers for breast cancer subtyping 27.

ALDH1 status changed from negative to positive after either PAM- or *invivo*Pen-treatment (Figure 6C).

## Discussion

Substances generated from *invivo*Pen are largely comprised of nitrogen oxides and hydroxide ions, with little helium ions, indicating that a large amount of nitrogens and oxygens as well as some water vapor from the air are effectively reassembled to form different reactive oxygen and nitrogen species (RONS) capable of conferring multi-modal efficacies.

The *invivo*Pen exhibited similar effects as PAM on suppressing cancer cell proliferation and migration, demonstrating the success of CAP ejection *in situ* without being clogged by the surrounding tissues through the use of *invivo*Pen. Interestingly, mice treated with *invivo*Pen delivered better viabilities and survival rates than those administrated with PAM, suggesting that direct CAP treatment *in vivo* could avoid any potential side effects caused by the cytotoxic substances generated from reactions between components in the medium and RONS. Also, it is indicative of a more stimulated immune system in mice by *invivo*Pen than PAM. CAP has been reported capable of stimulating the activities of immune cells such as macrophages^28^ and inducing immunogenic cell death^29^-^32^ that enable its killing effect on distant cancer cells. Whether direct *in situ* CAP ejection such as using *invivo*Pen can generate different *in vivo* effects on the immune system than PAM administration worths further investigations.

The animal blood test showed that after being exposed to CAP, either using PAM or *invivo*Pen, indexes on white blood cells such as lymphocyte, monocytes, neutrophil granulocyte, eosinophil granulocyte, and basophil granulocyte returned to normal, and *invivo*Pen showed higher efficacy in reducing monocyte and neutrophil granulocyte number than PAM. As a higher lymphocyte level is suggestive of a better immune response, and lower levels on monocytes, neutrophil granulocyte, eosinophil granulocyte, and basophil granulocyte implicate less inflammation, we would deduce from these results that physical plasma could boost animal immunity and confer anti-inflammation efficacy besides selectively killing cancer cells. Further, the slightly better performance observed on *invivo*Pen in returning indexes on white blood cells back to the healthy state indicates the advantage of direct* in vivo* treatment over indirect PAM in cancer control and the necessity of designing and fabricating *in vivo* plasma ejection sources translating physical plasma into clinics.

Indexes on red blood cells including red blood cell, average corpuscular volume, average corpuscular hemoglobin, average corpuscular hemoglobin concentration were not significantly altered between healthy and tumor-bearing mice, and physical plasma did not cause significant fluctuations to them, suggesting that red blood cells are not relevant indexes here and being exposed to physical plasma, either in the form of PAM or *invivo*Pen would not cause side effects on animal red blood cell levels.

Renal functionalities were dampened in mice inoculated with tumor cells, and both PAM and *invivo*Pen treatments could effectively rescue these experimental animals from renal dysfunctionality, suggesting the efficacy of physical plasma in cancer control. The liver functionality assay did not show significant alterations among healthy mice, mice carrying tumors and tumors receiving PAM or *invivo*Pen treatment overall, despite the large within-group variations that might be caused by the assay sensitivity to animal conditions at the time of test, further demonstrating the safety of physical plasma as an onco-therapy.

The cost of *invivo*Pen is lower than PAM as it does not need liquid as the media to confer CAP efficacy, and the cost of fabricating *invivo*Pen nozzle is equivalent to that of the needle used for PAM injection.

Both *invivo*Pen and PAM treatment could induce ER and HER2 expression, suggesting a state transition of tumors from the triple negative to the luminal-like phenotype after CAP treatment. Being a less malignant type of breast cancers with known targeted therapies (e.g. Tamoxifen and Herceptin), such tumor state transition not only partially explains the more favorable outcome of mice observed in the experiments but also implicates potential synergies between CAP and Tamoxifen and/or Herceptin in targeting TNBCs.

Though the expression of ALDH1, a stem cell marker 33, increased after *invivo*Pen or PAM treatment, ALDH1-high spots exhibited increased cell death, suggesting that cells with higher cancer stemness have higher sensitivities to CAP treatment. This is consistent with our observation that CAP could selectively target TNBC cells that have higher cancer stemness than the other breast cancer cells 24.

We present in this paper a novel *in situ* ejection source of CAP, namely *invivo*Pen, for animal experiments or human treatments if applied in clinics. It can deliver similar efficacies as PAM with reduced cost and improved safety in suppressing cancer cell proliferation and migration, and is more protective on the viabilities of tested mice. This proposed* invivo*Pen may serve as the prototype of the minimal invasive source for physical plasma ejection applied in clinics in the future.

## Supplementary Material

Supplementary tables.

## Figures and Tables

**Table 1:** Leukocyte components Analysis from the blood test.

	2 Weeks			4 Weeks					5 Weeks			
	Healthy	Tumor		Healthy	Tumor	PAM	invivoPen		Healthy	Tumor	PAM	invivoPen
**WBC**	5.71	6.48		5.07	9.92	4.51	5.33		1.92	12.09	2.37	2.51
**Neu**	1.44	2.14		1.48	7.39	1.59	1.61		0.70	10.2	1.12	1.06
**Lym**	3.58	3.53		3.11	1.29	2.57	3.24		1.027	0.70	0.98	1.27
**Mon**	0.46	0.57		0.32	0.85	0.23	0.29		0.09	0.76	0.19	0.11
**Eos**	0.19	0.16		0.16	0.29	0.11	0.16		0.09	0.34	0.07	0.065
**Bas**	0.04	0.08		0.01	0.1	0.01	0.037		0	0.09	0.0067	0.005

The unit is 109/L. WBC, Neu, Lym, Mon, Eos and Bas each represents white blood cell, neutrophil granulocyte, lymphocyte, monocytes, eosinophil granulocyte and basophil granulocyte number, respectively.

**Table 2:** Renal function analysis from the urine test.

	CREA-S(μmol/L)	UA(μmol/L)	UREA(μmol/L)
**Healthy1**	10.60	67.00	12.26
**Healthy2**	13.4	58.90	15.80
**Healthy3**	12.4	59.60	14.55
**Healthy4**	11.9	81.40	10.61
**Tumor1**	28.4	127.5	20.57
**Tumor2**	36.9	146.5	23.35
**PAM1**	21.4	94.00	12.60
**PAM2**	13.3	56.80	17.92
**PAM3**	12.7	77.90	14.53
**invivoPen1**	13.1	83.60	18.55
**invivoPen2**	22.2	112.6	17.98
**invivoPen3**	17.1	56.40	16.25
**invivoPen4**	17.4	68.90	21.02

CREA-S, UA and UREA each represents creatinine, uric acid and urea, respectively.
