# Supplementary material for: InvivoPen: A novel plasma source for in vivo cancer treatment
Source: J Cancer. 2020 Feb 10;11(8):2273–82. doi: 10.7150/jca.38613 (PMC7052936; doi:10.7150/jca.38613)
Supplement: Supplementary file 1 — Supplementary tables. [file jcav11p2273s1.pdf]

Supplementary Table 1. Erythrocyte components analysis from the blood test. RBC, MCV, MCH, MCHC, RDW-CV, RDW-SD and HCT each represents red blood cell, mean corpuscular volume, mean corpuscular hemoglobin, mean corpuscular hemoglobin concentration, RDW coefficient of variation, RDW standard deviation and hematocrit, respectively.

	**2 WEEKS**		**4 WEEKS**				**5 WEEKS**			
	**Healthy**	**Tumor**	**Healthy**	**Tumor**	**PAM**	**invivoPen**	**Healthy**	**Tumor**	**PAM**	**invivoPen**
RBC (10^{12}/L)	8.63	9.43	8.01	8.33	8.53	9.23	7.60	7.08	9.39	8.85
MCV (fL)	48.68	48.58	48.53	46.00	48.77	47.63	47.80	41.20	48.30	47.93
MCH (pg)	17.45	17.50	18.03	16.37	17.47	16.70	16.73	13.70	16.17	16.10
MCHC (g/L)	359.25	360.17	372.00	355.67	357.67	350.67	349.67	333.00	335.33	335.75
RDW-CV (%)	17.33	13.88	13.57	15.10	13.50	13.20	13.23	18.60	13.40	13.73
RDW-SD (fL)	36.73	29.90	29.23	31.33	29.40	27.83	27.67	33.30	28.53	29.08
HCT (%)	41.95	45.80	38.83	38.37	41.57	43.97	36.13	29.20	45.37	42.38

Supplementary Table 2. Liver function analysis from the urine test. ALT, AST, ALP and γ-GT each represents alanine aminotransferase, aspartate aminotransferase, alkaline phosphatase and γ- glutamyl transferase, respectively.

	ALT(U/L)	AST(U/L)	ALP(U/L)	γ-GT(U/L)
Healthy1	200.9	110.5	147.6	11.4
Healthy2	192.5	122	152.8	7.5
Healthy3	193.6	103.8	140	16
Healthy4	188.9	143.1	146.3	20.6
Tumor1	149.8	130.9	95.5	27.8
Tumor2	234.2	134.5	106.2	1.6
PAM1	192.5	170.5	131.3	6
PAM2	278.9	477.5	629.8	8.4
PAM3	207.2	113.7	143.9	10.1
invivoPen1	271.8	586.4	459.9	1.6
invivoPen2	313.6	319	131.7	16.5
invivoPen3	175.9	166.8	156.8	14.6
invivoPen4	243.4	158.6	126.4	10.8
